# Correlation between MBL2/CD14/TNF-α gene polymorphisms and susceptibility to spinal tuberculosis in Chinese population

**DOI:** 10.1042/BSR20171140

**Published:** 2018-02-02

**Authors:** Mingfeng Zheng, Shiyuan Shi, Wei Wei, Qi Zheng, Yifan Wang, Xiaozhang Ying, Di Lu

**Affiliations:** 1 Department of Orthopedics, Hangzhou Red Cross Hospital, Hangzhou, Zhejiang, China; 2 Department of Orthopedics, Tongde Hospital of Zhejiang Province, Hangzhou, Zhejiang, China

**Keywords:** Cluster of differentiation 14, Mannose-binding lectin, Spinal tuberculosis, Single nucleotide polymorphism, Tumor necrosis factor- α

## Abstract

Objective: The present study investigated the clinical significance of mannose-binding lectin 2 (MBL2), cluster of differentiation 14 (CD14) and tumour necrosis factor-α (TNF-α) gene polymorphisms in patients with spinal tuberculosis (TB) in Chinese population. Methods: A total of 240 patients with spinal TB were enrolled in the present study from May 2013 to August 2016 at Hangzhou Red Cross Hospital. A total of 150 age- and sex-matched healthy subjects were enrolled as controls. The genomic DNA was extracted from the peripheral blood of all subjects, and the MBL2, CD14 and TNF-α gene polymorphisms were detected by direct DNA sequencing. Results: (1) Compared with controls, patients with spinal TB exhibited a significantly higher frequency of the XY genotype at the −221G>C polymorphism as well as the Q allele and PQ genotype or an association with the QQ genotype at the +4C>T polymorphism in the MBL2 gene. (2) Compared with controls, patients with spinal TB exhibited a significantly higher frequency of the T allele and TT genotype or an association with the CT genotype at the −159C>T polymorphism in the CD14 gene. (3) Compared with controls, patients with spinal TB exhibited a significantly higher frequency of the T allele and the CT genotype or an association with the TT genotype at the TNF-857 polymorphism in the TNF-α gene. Conclusion: The −221G>C polymorphism of MBL2, the −159C>T polymorphism of CD14 and the TNF-857 polymorphism of TNF-α are risk factors for spinal TB and may be involved in the development of spinal TB in the Chinese population. These factors are indicators of susceptibility to spinal TB and require clinical attention.

## Introduction

Tuberculosis (TB) is an infectious disease caused by the bacterium *Mycobacterium tuberculosis* (MTB) [1]. An estimated 10.4 million people contracted TB in 2015, and it caused 1.8 million deaths. Therefore, TB is still a leading cause of death worldwide [2]. TB generally affects the lungs but can also affect other parts of the body, including the spine. Spinal TB is a common and dangerous extrapulmonary form of TB that accounts for 50% of all patients with bone or joint TB, and 10–43% of cases result in disability [3,4]. Unfortunately, the population susceptible to spinal TB has not been identified, and spinal TB remains an ongoing serious threat to public health with high mortality worldwide [5,6]. Previous studies suggested that host susceptibility genes may determine the response of an individual to *M. tuberculosis* infection, such as macrophage migration inhibitory factor, transforming growth factor-β (TGF-β) and interferon-γ (IFN-γ) [7,8]. However, the achievements of genetics studies have not yet advanced the prevention and treatment of spinal TB. Therefore, researchers need to widen their scope of investigation to encompass these practical considerations and finally discover the genetic markers for spinal TB that could be used in the clinic for the Chinese population.

Mannose-binding lectin 2 (MBL2) belongs to a family of proteins called the collectins, which possess both collagenous regions and lectin domains [9]. This protein consists of multimers of an identical polypeptide chain of 32 kDa. MBL2 plays an important role in host defence against pathogens [10]. Several groups have studied MBL2 genotypes and TB, following a suggestion that MBL2 deficiency might have had an evolutionary advantage by reducing the capacity of mycobacteria to invade macrophages in the absence of MBL2, leading to resistance to TB [11]. A study carried out in South Africa suggested that MBL-54 heterozygotes may have protection against tuberculous meningitis [12]. However, the association of MBL2 with spinal TB in the Chinese population has not been reported.

Cluster of differentiation 14 (CD14) is located on chromosome 5q31, which encodes a 55-kDa glycoprotein that is an important receptor for pattern recognition in the innate immunity system [13]. CD14 is a genetic factor for individual variant susceptibility, and its role has been investigated in many diseases, such as inflammatory bowel disease, *Helicobacter pylori* infection-related gastric carcinoma, asthma and TB [14]. Associations between CD14 polymorphisms and risk of TB have been found in some, but not all, studies [15,16]. This inconsistency may be the result of relatively small sample sizes and different ethnicities.

Tumour necrosis factor-α (TNF-α) is produced by macrophages, dendritic cells and T cells when stimulated or infected with *M. tuberculosis* [17,28]. In mice deficient in TNF-α or the 55-kDa TNF receptor, *M. tuberculosis* infection resulted in rapid death, with a higher bacterial burden than that observed in control mice [19]. Moreover, the clinical course of human TB shows that high levels of TNF-α have been associated with clinical decline in patients with TB, and higher secretion of TNF-α in humans could be associated with the haematogenous dissemination of *M. tuberculosis* to other organs [20,21]. The TNF-α −308 G/A polymorphism was found to protect against TB in Sicily, and the −308A-238G haplotype was protective in Colombia [22]. However, the association of TNF-α with spinal TB in the Chinese population has not been reported.

MBL2, CD14 and TNF-α are three crucial genes playing important roles in immune inflammation. The roles of these three gene polymorphisms in the susceptibility to spinal TB have not been well addressed. In the present study, we investigated the genotype and allele frequencies of MBL2, CD14 and TNF-α polymorphisms in 240 patients with spinal TB and 150 healthy controls. Furthermore, we analysed the associations of the genotypes of these genes with susceptibility to spinal TB and provided a basis for clinical individualized medicine.

## Materials and methods

### Subjects

A total of 240 patients diagnosed with spinal TB were enrolled in the present study from May 2013 to August 2016 at Hangzhou Red Cross Hospital. Spinal TB was diagnosed according to findings from a medical history of tuberculosis, laboratory tests such as a positive skin test and elevated erythrocyte sedimentation rate (ESR), histological analysis of the pathogen, computed tomography (CT) and magnetic resonance imaging (MRI). Patients with pulmonary TB and other extrapulmonary forms of TB were excluded by chest X-ray and B ultrasonography. The spinal TB patients with comorbid disorders or other complications, such as rheumatoid arthritis, congenital cervical anomalies, trauma, prior spinal cervical surgery, HIV-positive or ankylosing spondylitis were excluded from the present study. Then, 150 age- and sex-matched healthy subjected were enrolled as controls. The study protocol was approved by the Ethics Committees at Hangzhou Red Cross Hospital. Signed informed consent was obtained from all patients, and the present study was carried out in compliance with the Helsinki Declaration.

### Genotyping and single nucleotide polymorphism

Five millilitres of peripheral blood was collected into EDTA tubes. Genomic DNA was isolated from white blood cells using a Qiagen Genomic DNA Isolation Kit (51104, Qiagen, Valencia, CA, U.S.A.). The DNA polymorphisms in the MBL2, CD14 and TNF-α genes were detected by direct DNA sequencing for all samples by the Sanger sequencing method. The MBL2, CD14 and TNF-α genes were amplified by polymerase chain reaction (PCR) using the forward and reverse primers shown in Table 1.

**Table 1:** The primer sequences for MBL2, CD14 and TNF-α gene amplification

Gene/polymorphism		Primer
TNF-238/244	Forward	5′-CCTCAAGGACTCAGCTTTCGT-3′
	Reverse	5′-ACACTCCCCATCCTCCCACATC-3′
TNF-863	Forward	5′-GGCTCTGAGGAATGGGTTAC-3′
	Reverse	5′-CCTCTACATGGCCCTGTCTAC-3′
TNF-857	Forward	5′-GGCTCTGAGGAATGGGTTAC-3′
	Reverse	5′-CCTCTACATGGCCCTGTCTAC-3′
TNF-308	Forward	5′-AGGCAATAGGTTTTGAGGGCCAT-3′
	Reverse	5′-TCCTCCCTGCTCCGATTCCG-3′
MBL2	Forward	5′-GCCAGTGGTTTTTGACTCAC-3′
	Reverse	5′-GGAGTATAGGGGTCCGTCA-3′
CD14 −159C>T	Forward	5′-GCCAACAGATGAGGTTCACA-3′
	Reverse	5′-GTTCGACCCCAAGACCCTAC-3′

### Statistical analysis

Quantitative data were analysed using the Statistical Package for the Social Sciences, version 20.0 (SPSS 20.0). The frequency of various genotypes in the controls and patients was subjected to Hardy–Weinberg equilibrium analysis. The qualitative data were expressed as absolute numbers and/or percent values. Genotypes and allele frequencies between patients and controls were compared using a χ^2^ test. The adjusted odds ratio (OR), 95% confidence interval (CI) and adjusted *P* value were calculated. The results were considered statistically significant at *P*<0.05.

## Results

### MBL2 genotype distributions and allele frequencies

**Figure 1:**
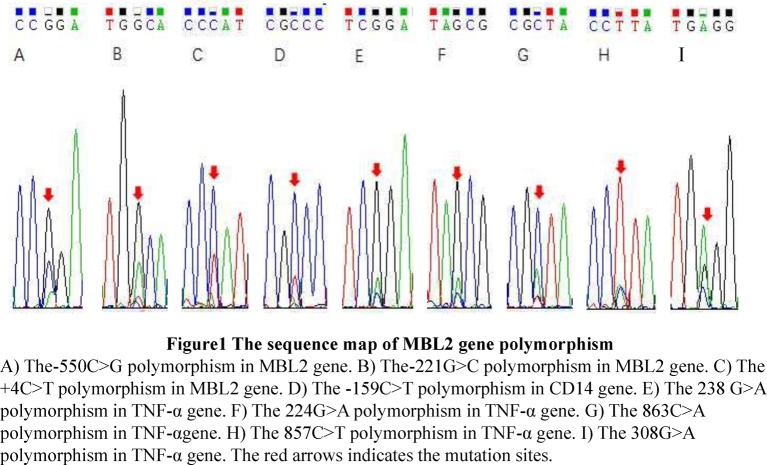
The sequence map of MBL2 gene polymorphisms (**A**) The −550C>G polymorphism in the MBL2 gene. (**B**) The −221G>C polymorphism in the MBL2 gene. (**C**) The +4C>T polymorphism in the MBL2 gene. (**D**) The −159C>T polymorphism in the CD14 gene. (**E**) The 238 G>A polymorphism in the TNF-α gene. (**F**) The 224G>A polymorphism in the TNF-α gene. (**G**) The 863C>A polymorphism in the TNF-α gene. (**H**) The 857C>T polymorphism in the TNF-α gene. (**I**) The 308G>A polymorphism in the TNF-α gene.

Genotypes and allele frequencies at the MBL2 genetic locus are shown in Table 2. The genotype frequencies for all polymorphisms in the MBL2 gene were Hardy–Weinberg balanced (*P*>0.05). In the MBL2 gene, the −550C>G, −221G>C and +4C>T polymorphisms were measured. Spinal TB patients showed no significant differences from the control subjects in genotype and allele frequencies of the −550C>G polymorphism. In contrast, although there was no significant difference in allele frequencies of X and Y between spinal TB patients and controls for the −221G>C polymorphism, the frequency of XY genotypes with the −221G>C polymorphism was significantly higher in spinal TB patients than that in controls (*P*<0.05). These results showed that with YY, XX and YX + XX genotypes as references, individuals with the XY genotype at −221G>C had a higher risk for spinal TB. For the +4C>T polymorphism, the frequency of the Q allele was significantly higher in spinal TB patients than in controls (*P*<0.05), and the frequency of the PQ genotype and its association with the QQ genotype were also significantly higher in spinal TB patients than in controls (*P*<0.05). These results showed that individuals with the Q allele in the +4C>T locus of the MBL2 gene had a higher risk for spinal TB, and the PQ genotype or the association with the QQ genotype also increased the risk of spinal TB. The DNA sequencing results are shown in Figure 1A–C.

**Table 2:** The genotypic and allelic frequencies of MBL2 gene polymorphisms between spinal tuberculosis and controls

	Patients (*n*=240) *N* (%)	Control (*n*=150) *N* (%)	Adjusted *P*	Adjusted OR	95% CI
**−550C>G**					
Genotype					
HH	14 (5.83%)	20 (13.33%)	Ref	1.00	Ref
HL	120 (50.00%)	61 (40.67%)	0.152	1.98	0.74–5.14
LL	106 (44.17%)	69 (46.00%)	0.071	2.31	0.91–5.87
HL + LL	226 (94.17%)	130 (86.67%)	0.081	2.16	0.87–5.29
Allele					
H	130 (27.08%)	98 (32.67%)	Ref	1.00	Ref
L	350 (72.92%)	202 (67.33%)	0.074	1.40	0.97–2.01
**−221G>C**					
Genotype					
YY	149 (62.08%)	112 (74.67%)	Ref	1.00	Ref
XY	84 (35.00%)	33 (22.00%)	0.021	1.87	1.13–4.15
XX	7 (2.92%)	7 (4.67%)	1	0.97	0.20–4.18
YX + XX	67 (27.92%)	39 (26.00%)	0.062	1.71	0.95–3.01
Allele					
Y	409 (85.21%)	256 (85.33%)	Ref	1.00	Ref
X	71 (14.79%)	14 (14.67%)	0.109	1.47	0.87–2.27
**+4C>T**					
Genotype					
PP	144 (60.00%)	110 (73.33%)	Ref	1.00	Ref
PQ	82 (34.17%)	34 (22.67%)	0.014	1.91	1.12–6.31
QQ	14 (5.83%)	6 (4.00%)	0.365	1.79	0.51–3.54
PQ + QQ	96 (40.00%)	40 (26.67%)	0.022	1.85	1.07–3.24
Allele					
P	330 (7.08%)	253 (84.33%)	Ref	1.00	Ref
Q	110 (22.92%)	47 (15.67%)	0.012	2.65	1.09–4.51

### CD14 genotype distributions and allele frequencies

Genotypes and allele frequencies at the CD14 genetic locus are shown in Table 3. The genotype frequencies for all polymorphisms in the CD14 gene were Hardy–Weinberg balanced (*P*>0.05). In the CD14 gene, the −159C>T polymorphism was measured. In the −159C>T polymorphism, the frequency of the T allele was significantly higher in spinal TB patients than in controls (*P*<0.01), and the frequency of the TT genotype and the association with the CT genotype were also significantly higher in spinal TB patients than in controls (*P*<0.05). These results showed that patients with the T allele at the −159C>T locus of the CD14 gene had a higher risk for spinal TB, and the TT genotype or an association with the CT genotype increased the risk of spinal TB. The DNA sequencing results are shown in Figure 1D.

**Table 3:** The genotypic and allelic frequencies of CD14 gene polymorphisms between spinal tuberculosis and controls

	Patients (*n*=240) *N* (%)	Control (*n*=150) *N* (%)	Adjusted *P*	Adjusted OR	95% CI
**−159C>T**					
Genotype					
CC	36 (15.00%)	44 (18.33%)	Ref	1.00	Ref
CT	132 (55.00%)	81 (33.75%)	0.512	1.05	0.65–1.64
TT	72 (30.00%)	25 (10.42%)	0.021	1.68	1.02–3.58
CT + TT	204 (85.00%)	106 (44.17%)	0.032	2.10	1.09–3.85
Allele					
C	204 (42.50%)	84 (56.00%)	Ref	1.00	Ref
T	276 (57.50%)	66 (44.00%)	0.001	1.97	1.24–3.42

**Table 4:** The genotypic and allelic frequencies of TNF-α gene polymorphisms between spinal tuberculosis and controls

	Patients (*n*=240) *N* (%)	Control (*n*=150) *N* (%)	Adjusted *P*	Adjusted OR	95% CI
**TNF-238**					
Genotype					
GG	217 (90.42%)	143 (95.33%)	Ref	1.00	Ref
GA	23 (9.58%)	7 (4.67%)	0.525	1.14	0.79–1.54
AA	0 (0)	0 (0)	NA	NA	NA
GA + AA	23 (9.58%)	7 (4.67%)	0.154	1.62	0.95–1.67
Allele					
G	457 (95.21%)	293 (97.67%)	Ref	1.00	Ref
A	23 (4.79%)	7 (2.33%)	0.512	1.75	0.80–1.58
**TNF-224**					
Genotype					
GG	240 (100%)	150 (100%)	NA	NA	NA
GA	0 (0)	0 (0)	NA	NA	NA
AA	0 (0)	0 (0)	NA	NA	NA
GA + AA	0 (0)	0 (0)	NA	NA	NA
Allele					
G	480 (100%)	300 (100%)	NA	NA	NA
A	0 (0)	0 (0)	NA	NA	NA
**TNF-863**					
Genotype					
CC	178 (74.17%)	108 (72.00%)	Ref	1.00	Ref
CA	54 (22.50%)	42 (28.00%)	0.254	1.21	0.77–1.04
AA	8 (3.33%)	0 (0)	0.375	1.14	0.75–1.01
CA + AA	62 (25.83%)	42 (28.00%)	0.275	1.17	0.78–1.24
Allele					
C	410 (85.42%)	258 (86.00%)	Ref	1.00	Ref
A	70 (14.58%)	42 (14.00%)	0.385	1.05	0.81–0.94
**TNF-857**					
Genotype					
CC	132 (55.00%)	172 (84.67%)	Ref	1.00	Ref
CT	108 (45.00%)	26 (17.33%)	<0.001	8.85	2.42–30.84
TT	0 (0)	3 (2.00%)	0.245	2.35	0.21–3.54
CT + TT	108 (45.00%)	29 (19.33%)	<0.001	9.87	2.55–34.85
Allele					
C	372 (77.50%)	268 (89.33%)	Ref	1.00	Ref
T	108 (22.50%)	32 (10.67%)	0.001	7.54	2.54–25.87
**TNF-308**					
Genotype					
GG	21790.42%)	127 (84.67%)	Ref	1.00	Ref
GA	23 (9.58%)	23 (15.33%)	0.412	1.07	0.86–1.05
AA	0 (0)	0 (0)	NA	NA	NA
GA + AA	23 (9.58%)	23 (15.33%)	0.211	1.54	0.95–1.38
Allele					
G	457 (95.21%)	277 (92.33%)	Ref	1.00	Ref
A	23 (4.79%)	23 (7.67%)	0.387	1.41	0.87–1.27

### TNF-α genotype distributions and allele frequencies

Genotypes and allele frequencies for the TNF-α genetic locus are shown in Table 4. The genotype frequencies for all polymorphisms in the TNF-α gene were Hardy–Weinberg balanced (*P*>0.05). In the TNF-α gene, the TNF-238, TNF-224, TNF-863, TNF-857 and TNF-803 polymorphisms were measured. Spinal TB patients showed no significant differences from the control subjects in genotype and allele frequencies for the TNF-238, TNF-863 or TNF-308 polymorphisms. For the TNF-857 polymorphism, the frequency of the T allele was significantly higher in spinal TB patients than in controls (*P*<0.001), and the frequency of the CT genotype and the association with the TT genotype were also significantly higher in spinal TB patients than in controls (*P*<0.001). These results showed that patients with the T allele at the TNF-857 locus of the TNF-α gene had a higher risk for spinal TB, and the CT genotype or the association with the TT genotype increased the risk of spinal TB. The DNA sequencing results are shown in Figure 1E– I.

## Discussion

Host genotype is increasingly found to be associated with variations in host susceptibility and outcomes in *M.* tuberculosis infection. Studies have shown that the incidence of TB in identical twins was significantly higher than that of fraternal twins, which suggests that genetic factors correlate with susceptibility to TB [23]. With the advances in sequencing technologies, a number of case–control studies, such as genome-wide association studies (GWAS), have been performed to explore genetic determinants of TB and have had some positive results. Although spinal TB occurs in fewer than 1% of TB patients, it is a common and dangerous type of extrapulmonary TB accounting for 50% of all patients with bone or joint TB. However, only a few studies have focused on the genetic susceptibility to spinal TB and the genes associated with susceptibility to spinal TB have not been identified. In the present study, we identified the roles of MBL2, CD14 and TNF-α gene polymorphisms in the susceptibility to spinal tuberculosis in a Chinese population.

MBL2 plays an important role in host defence against pathogens [9]. Several groups have studied MBL2 genotypes and TB, following a suggestion that MBL2 deficiency might reduce the capacity of mycobacteria to invade macrophages, thereby leading to resistance to TB. A study carried out in South Africa suggested that MBL-54 heterozygotes may have protection against tuberculous meningitis [24]. Therefore, in the present study, we analysed the −550C>G, −221G>C and +4C>T polymorphisms of the MBL2 gene, whose association with TB has been reported [25,26], but its role in spinal TB has not previously been explored. Our results showed significantly higher frequencies of the PQ genotype or an association with the QQ genotype and Q allele in the +4C>T polymorphism among patients with spinal TB than in healthy controls. In addition, the frequencies of the XY genotype were significantly higher than in controls. These results suggested that MBL2 gene +4C>T locus Q allele carriers are susceptible to spinal TB. Increased MBL2 combined with *M. tuberculosis* might act as an opsonin and promote phagocytosis, thereby affecting the inflammatory response to spinal TB.

CD14 has been identified as a genetic factor for individual variant susceptibility, and its role has been investigated in many diseases, such as inflammatory bowel disease, asthma and TB [27, 28]. A single nucleotide polymorphism at −159C>T in the promoter of CD14 can influence CD14 expression, which is thought to be associated with TB development [29]. Many studies have therefore examined any possible correlation, but the results are still inconclusive. A study in Koreans found that the −159T allele had stronger promoter activity and was a risk factor for TB [30]. However, few studies have focused on any possible correlation between CD14 gene polymorphisms and the susceptibility to extrapulmonary TB, especially spinal TB. Our results indicated that patients with the T allele had a greater likelihood of spinal TB than those with the C allele and that TT carriers had prominent spinal TB susceptibility compared with individuals with CC and C alleles in the overall analysis. Previous studies have provided some clues to understanding these results [31,32]. The CD14-159T allele could reduce the affinity between the CD14 promoter and transcriptional inhibitor SP3, enhance transcriptional activity and increase the levels of CD14 expression, particularly circulating sCD14 levels. sCD14 might compete with mCD14-mediated uptake of *M. tuberculosis* into human microglia [33].

TNF-α is produced by macrophages, dendritic cells and T cells when stimulated or infected with *M. tuberculosis* [34]. In mice deficient in TNF-α or the 55-kDa TNF receptor, *M. tuberculosis* infection resulted in rapid death, with a higher bacterial burden than that observed in control mice [35]. Moreover, in the clinical course of human TB, high levels of TNF-α have been associated with clinical decline in patients with TB, and higher secretion of TNF-α in humans could be associated with the haematogenous dissemination of *M. tuberculosis* to other organs [36,37]. The TNF-α −308 G/A polymorphism was found to protect against TB in Sicily and the −308A-238G haplotype was protective in Colombia [38]. However, any association of TNF-α with spinal TB in the Chinese population has not been reported. Our results indicated that patients with a T allele at the TNF-857 locus of the TNF-α gene had a higher risk for spinal TB, and the CT genotype or an association with the TT genotype increased the risk of spinal TB. These results might be related to the immune response to spinal TB because TNF-α is one of the important immune inflammatory factors [17,18]. Mutations in the TNF-α gene may affect transcriptional activation and alter the levels of TNF-α production, further affecting the immune response to spinal TB.

The development of spinal TB is the result of a complex interaction between the host and pathogen influenced by environmental factors. Susceptibility to spinal TB in humans appears to be highly polygenic with many loci implicated but only a minority of these have been convincingly proven. Our results suggest that the MBL2 gene +4C>T locus Q allele, the CD14 gene T allele, the TNF-α gene T allele, the MBL2 +4C>T PQ genotype, the CD14-159C>T TT genotype, and the TNF-857 CT genotype are associated with increased spinal TB susceptibility in the Chinese population. These findings may help to identify high-risk populations and create more effective prevention and treatment strategies. The present study highlights the potential role of immunogenetics in the clinical management of spinal TB. Furthermore, we hope that in the near future, genetic studies and our results may provide a basis for clinical individualized medicine.

## Abbreviations

CD14: cluster of differentiation 14
CI: confidence interval
CT: computed tomography
ESR: elevated erythrocyte sedimentation rate
IFN-γ: interferon-γ
MBL2: mannose-binding lectin 2
MTB: *Mycobacterium tuberculosis*
OR: odds ratio
TB: spinal tuberculosis
TGF-β: transforming growth factor-β i
TNF-α: tumour necrosis factor-α
